# PLK1-mediated phosphorylation of PPIL2 regulates HR *via* CtIP

**DOI:** 10.3389/fcell.2022.902403

**Published:** 2022-08-25

**Authors:** Zhiyu Qiu, Shuailin Hao, Shikai Song, Ruiling Zhang, Tingyu Yan, Zhifang Lu, Hailong Wang, Zongchao Jia, Jimin Zheng

**Affiliations:** ^1^ College of Chemistry, Beijing Normal University, Beijing, China; ^2^ Beijing Key Laboratory of DNA Damage Response and College of Life Sciences, Capital Normal University, Beijing, China; ^3^ Department of Biomedical and Molecular Sciences, Queen’s University, Kingston, ON, Canada

**Keywords:** biochemistry, cell biology DNA damage, DNA double-strand break, CtIP, PPIL2, PLK1, ZNF830

## Abstract

Homologous recombination (HR) is an error-free DNA double-strand break (DSB) repair pathway, which safeguards genome integrity and cell viability. Human C-terminal binding protein (CtBP)—interacting protein (CtIP) is a central regulator of the pathway which initiates the DNA end resection in HR. Ubiquitination modification of CtIP is known in some cases to control DNA resection and promote HR. However, it remains unclear how cells restrain CtIP activity in unstressed cells. We show that the ubiquitin E3 ligase PPIL2 is recruited to DNA damage sites through interactions with an HR-related protein ZNF830, implying PPIL2’s involvement in DNA repair. We found that PPIL2 interacts with and ubiquitinates CtIP at the K426 site, representing a hereunto unknown ubiquitination site. Ubiquitination of CtIP by PPIL2 suppresses HR and DNA resection. This inhibition of PPIL2 is also modulated by phosphorylation at multiple sites by PLK1, which reduces PPIL2 ubiquitination of CtIP. Our findings reveal new regulatory complexity in CtIP ubiquitination in DSB repair. We propose that the PPIL2-dependent CtIP ubiquitination prevents CtIP from interacting with DNA, thereby inhibiting HR.

## Introduction

DNA double-strand breaks (DSBs) are considered the most dangerous of all DNA lesions, and can be caused by a variety of endogenous factors, including DNA replication errors, reactive oxygen species; and exogenous factors, including X-ray exposure and chemical treatments. DSBs occur frequently in daily life, and if left unrepaired, the broken chromosome can be lethal to the cell. To maintain genomic stability, cells have developed a comprehensive DNA repair system and cell cycle checkpoints to ensure that the DNA damage is repaired before the cell cycle resume.

Repair of DSBs in mammalian cells occurs mainly through either classical nonhomologous end-joining (C-NHEJ), or homologous recombination (HR) (Lieber 2010; Moynahan and Jasin 2010). The first key step in HR is 5′–3′ DNA resection at the DSB site, which generates an extension of 3′-single-stranded DNA (ssDNA) (Symington 2014; Vitor et al., 2020). This process is controlled by the cell cycle and involves multiple factors, including the MRN complex (Mre11-Rad50-NBS1), CtIP, Exo1, DNA2, and BLM (Symington and Gautier 2011). Replicating protein A (RPA) then binds and accumulates on the ssDNA resection product, initiating Rad51 recombinase nuclear-protein filament formation (Lopez-Saavedra et al., 2016). Next, the filaments search for homology and perform strand invasion to complete HR (San Filippo et al., 2008). HR depends on the identical sister chromatid as a template, and is thus usually error-free and limited to the S/G2 phase of the cell cycle (Mladenov et al., 2013).

C-NHEJ is a more error-prone DSB repair mechanism that operates throughout all phases of the cell cycle (Mladenov, Magin, Soni and Iliakis 2013). Although C-NHEJ can introduce deletion or insertion mutations, C-NHEJ is a rapid repair pathway that is critical to cells coping with acute DNA damage. In constrast, the alternative NHEJ (A-NHEJ) pathway is usually seen as a backup repair pathway that works through microhomology-mediated end joining (MMEJ). MMEJ does not require Ku, but depends on 5′ to 3′ resection factors such as the MRN complex and CtIP (Seol et al., 2018).

CtIP is a central regulator that initiates DNA end resection and HR. CtIP was first identified as an interacting protein of the transcriptional repressor carboxy-terminal binding protein (CtBP) (Makharashvili et al., 2014), and CtIP has roles in multiple cellular processes, including cell cycle regulation and tumorigenesis (Wong et al., 1998). CtIP interacts with the MRN complex and BRCA1, and is involved in DNA resection and repair (Chen et al., 2008; You et al., 2009). CDK phosphorylation of CtIP induces the association of CtIP with the NBS1 FHA and BRCT domains to promote the initial stage of resection (Wang et al., 2013). At the beginning of end resection, Mre11 and CtIP work together to generate limited lengths of ssDNA at DNA damage sites (Takeda et al., 2007; Paull 2010). The length of ssDNA produced at this stage is very limited and not enough to promote HR. After MRN-CtIP-mediated end resection is initiated, Exo1 is recruited to the DSB site by the MRN complex and activates Exo1 and DNA2 nuclease activity to participate in long-range DNA end resection (Zhou et al., 2015; Anand et al., 2016; Oh et al., 2016; Levikova et al., 2017). DNA helicase BLM is also necessary for DNA2-mediated extensive end resection (Levikova et al., 2013). Multiple studies have shown that DNA2 cleaves ssDNA through the DNA unwinding activity of BLM during the long-range DNA end resection (Nimonkar et al., 2011; Sturzenegger et al., 2014; Pinto et al., 2016). CtIP also interacts with BLM and enhances long-range resection to promote HR (Daley et al., 2017).

Several studies have revealed that post-translational modifications control the activity or expression of CtIP. Ubiquitination plays multiple roles in DNA damage signalling and has been shown to regulate HR and NHEJ, thus ensuring appropriate DSB repair. Ubiquitination is a common and highly conserved protein modification in which ubiquitin (Ub) is added to its substrate through a cascade reaction that targets proteins for regulation and degradation in eukaryotes. There have been reports of several E3 ubiquitin ligases interacting with and ubiquitinating CtIP to alter its function. CtIP is ubiquitinated by APC/CCdh1, PIN1, and CUL3-KLHL15, which promotes the degradation of CtIP through the ubiquitin degradation pathway to inhibit its DNA resection function (Steger et al., 2013; Lafranchi et al., 2014; Ferretti et al., 2016). In addition, CtIP can also be ubiquitinated by BRCA1 and RNF138 to promote its recruitment to DNA damage sites and improve HR repair efficiency (Yu et al., 2006; Schmidt et al., 2015). Although there are some reports that ubiquitination of CtIP can promote its DNA resection activity, other studies point out that deubiquitinating enzymes can promote the activity of CtIP, such as USP52 (Gao et al., 2020). How and if CtIP activity is supressed in unstressed cells and activated when needed remains elusive. Therefore, the further research into the mechanism and inhibitory effects of CtIP ubiquitination is needed.

The previous studies have also shown the modified CtIP can be recruited to DNA damage sites, which may involve the interaction of CtIP with other repair factors, such as ZNF830. ZNF830 also known as ccdc16 or omcg1, is a nuclear zinc finger protein that ineracts directly with CtIP to promote DNA end resection and HR repair (Chen et al., 2018). ZNF830 has been shown to be essential to genomic integrity, as the deletion of mouse derived ZNF830-omcg1 in mouse embryonic fibroblasts causes an accumulation DNA double strand breaks, resulting in the activation of DNA damage checkpoints and the formation of highly stable DNA-RNA heteroduplexes (Houlard et al., 2011). ZNF830 binds to DNA through its N-terminal ZNF domain, and ATM/ATR kinase phosphorylates its S362 site, which promotes recruitment to the DSB site (Chen et al., 2018). In this study, we found that PPIL2, a ubiquitin E3 ligase, interacts with ZNF830 and is recruited to DNA damage sites, indicating PPIL2’s involvement in DSB repair. Knockdown of PPIL2 promotes HR, which seems to depend on CtIP. Other experiments demonstrate that PPIL2 could associate with CtIP and ubiquitinate CtIP at the K426 site to inhibite its activity in HR, representing a new ubiquitination site.

In addition, we found that PLK1 phosphorylates PPIL2 at 14 different sites. PLK1 (polo-like kinase 1) is a serine/threonine kinase that plays an important role in cell cycle processes including mitotic entry, centrosome maturation, microtubule nucleation, chromosome segregation, mitotic exit and cytokinesis (Colicino and Hehnly 2018). In the G2 phase, PLK1 is dephosphorylated in an ATM-Chk1-dependent manner, which inhibits its kinase activity. Therefore, PLK1 is downregulated until the DNA damage response (DDR) is completed, at which time the activation of PLK1 acts as a checkpoint regulator, allowing the cells to enter M phase (Lee et al., 2010). Phosphorylation of PPIL2 by PLK1 suppresses its interaction with CtIP and reduces PPIL2 ubiquitination of CtIP. When DSBs occur, the interaction of PPIL2 with CtIP and the ubiquitination of CtIP are reduced. Therefore, the ubiquitination of CtIP by PPIL2 at CtIP K426 represents a new regulatory complexity that inhibits CtIP activity whereby PPIL2 can inhibit HR. This regulatory complexity may act as an important regulator that helps suppress CtIP activity in unstressed cells, and PPIL2 may also be recruited to DSB to ubiquitinate CtIP and displace CtIP from DSBs after the resection in HR.

## Materials and methods

### Plasmid construction and shRNA sequencing

pcDNA3 Flag-CtIP and fragments and HA-Ub were provided by Hailong Wang (Capital Normal University, Beijing). PPIL2, ZNF830, and BLM cDNA was generated *via* PCR and ligated into the pcDNA3 vector (Invitrogen) containing a three N-terminal Flag tag and His tag. EGFP-PPIL2 was constructed using the EGFP-C1 expression vector (Clontech). HA-Ub K48/K63/K48R/K63R and Flag-CtIP K426A were constructed using the QuikChange Site-directed Mutagenesis Kit (Stratagene).

shRNA sequences for CtIP 5′-GAG​CAG​ACC​UUU​CUC​AGU​AUA-3′ have been previously described (Truong et al., 2013). shRNA sequencs for PPIL2 is 5′-GAA​ACG​UGA​UGA​AGA​AUU​GAG​A-3’. shRNA sequencs for ZNF830 is 5′-UAA​CCG​GAG​UGU​UAC​ACA​G-3’.

### Cell culture, transient transfections, and drug treatment

293T and U2OS cells were cultured at 37°C in a humidified atmosphere with 5% CO_2_ in DMEM (Gibco) supplemented with 10% foetal bovine serum (FBS; Sigma) and 1% penicillin/streptomycin (Corning).

DNA constructs were transiently transfected with PEI (Polysciences) for 4 h and after 48 h of transfection they were treated as indicated. The cells were treated with MG132 (20 μM, 3 h; Sigma), CPT (2 μM, 1 h; Sigma) or ATM inhibitor KU-55933 (20 mM, 2 h; Sigma).

### Immunoblotting and immunoprecipitation

Whole-cell lysis, immunoblotting and immunoprecipitation were performed as previously described (Wang et al., 2018). Anti-ZNF830 (HPA027211), anti-PPIL2 (HPA035344), anti-FLAG M2 (F1804) and anti-β-actin (A5441) were purchased from Sigma. Anti-PLK1 (sc-17783) and anti-CtIP (sc-48415) were purchased from Santa Cruz. Anti-HA (A190–208A) and anti-pH3S10 (A301-844A) were purchased from Bethyl. Anti-His (ab18184) and anti-GST (ab111947) were purchased from abcam.

### Chromatin immunoprecipitation assay

ER-*Asi*SI-expressing U2OS cells were treated with 4-OHT (300 nM) for 4 h. ChIP assay was performed as previously described (Tyteca et al., 2006). Chromatin was immunoprecipitated with Flag M2 beads (A2220; Sigma). The immunoprecipitated DNA and input DNA were analyzed by qPCR, using the following primers: F: 5′-GAT​TGG​CTA​TGG​GTG​TGG​AC-3′; R: 5′ -CAT​CCT​TGC​AAA​CCA​GTC​CT-3 ′. The IP efficiency was calculated as the percent of the input DNA immunoprecipitated.

### Expression of recombinant protein, purification and GST-pulldown

His-tagged PLK1 or PPIL2 fragments were generated using the pET28b (Invitrogen) and GST-tagged ZNF830 fragments were generated using the pGEX6T-1 (GE Healthcare) system. His or GST-tagged recombinant protein were expressed in *E. coli* (BL21). After cell lysis, proteins were purified using Ni-NTA (Qiagen) or glutathione-Sepharose 4B (GE Healthcare) according to the manufacturer’s recommendations.

Full length or fragments GST-tagged ZNF830 and His-tagged PPIL2 were expressed in *E. coli* and GST-pulldown assay were performed as previously described (Wu et al., 2019).

### Laser-induced micro-radiation and live cell imaging

U2OS cells expressing the EGFP-tagged PPIL2 were cultured in DMEM with 10% FBS in a sterile glass-bottom dish (MatTek). DSBs were induced by local irradiating with a 365 nm pulsed nitrogen ultraviolet laser (16 Hz pulse, 41% laser output) on the nucleus of living cells and generated by the Micropoint System (Andor). Time-lapse images of live cells were captured on a Nikon A1 confocal imaging system directly coupled to the Micropoint system.

### HR, NHEJ, and MMEJ measurement

U2OS cells carrying an EGFP-HR, EGFP-NHEJ or EGFP-MMEJ reporter (Wang et al., 2012) lentivirus with the I-sce1 gene were infected with the reporter system for 16 h. Cells were collected after 48 h, and a CytoFLEX flow cytometer was used for detection with the analysis software CytExpert.

### *In vitro* kinase assay

*In vitro* analysis of PLK1 phosphorylated PPIL2 was preformed using WT or kinase dead (KD) mutant His-PLK1 expressed in and purified from *E. coli* BL21 cells. His-PLK1 WT or KD were incubated with purified PPIL2 in kinase buffer [25 mM HEPES buffer (pH 7.4), 50 mM NaCl, 1 mM Na_3_VO_4_, 10 mM MgCl_2_, 1 mM DTT, 10 μM ATP, and 10 μCi g-^32^P-ATP]. The kinase reaction was carried out at 30°C for 30 min. The reaction was stopped by boiling, and the cells were analysed by SDS–PAGE and autoradiography.

### Mass spectrometry

Ubiquitination sites were mapped *via* mass spectrometry. Flag-CtIP, His-PPIL2, and HA-Ub were coexpressed in 293T cells for 4 h, then MG132 was added for 3 h before harvesting the cells and lysed them in NETN buffer [20 mM Tris–HCl (pH 7.5), 150 mM NaCl, 1 mM EDTA, 0.5% NP-40] containing a protease inhibitor cocktail (PIC, Roche). The cell lysate was centrifuged, and the supernatant was added to anti-Flag M2-conjugated agarose beads (Sigma). After incubating overnight at 4°C, the beads were centrifuged and washed thoroughly in NETN buffer. Subsequently, Flag-CtIP was eluted with NETN buffer containing FLAG peptide and analysed by Liquid Chromatography-Mass Spectrometry.

## Results

### PPIL2 is recruited to DNA damage sites

**FIGURE 1:**
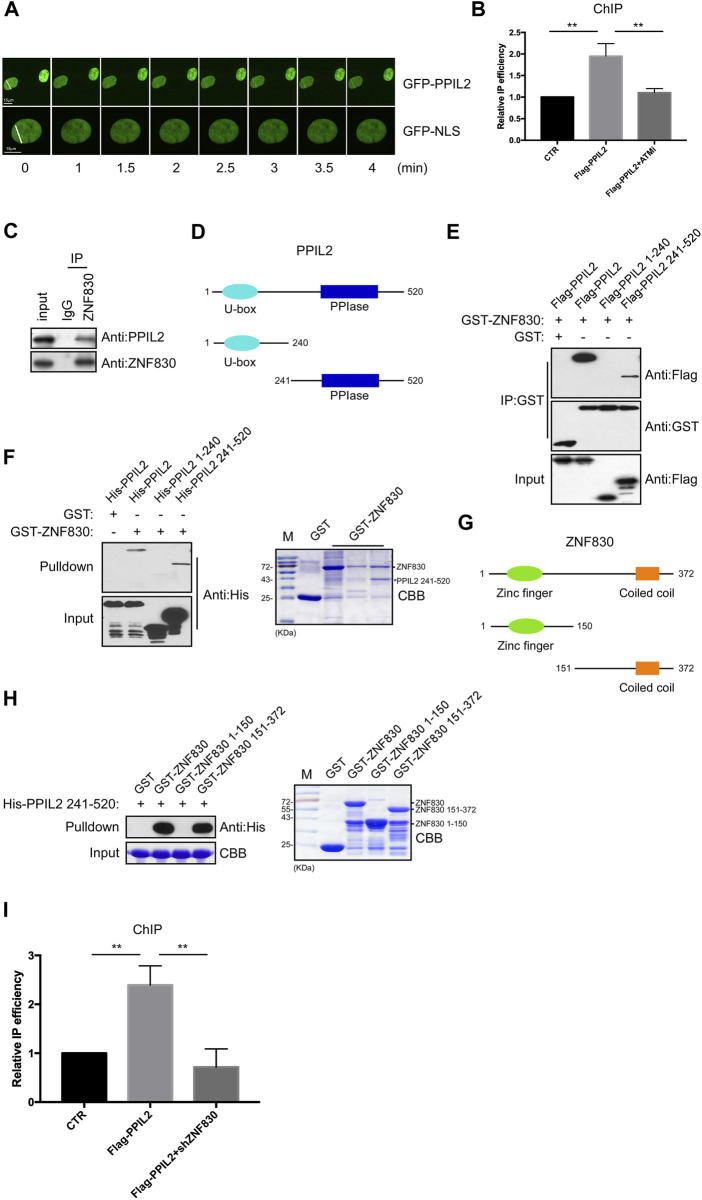
PPIL2 is recruited to DNA damage sites and interacts with ZNF830. **(A)** Live-cell imaging of recruitment showed that EGFP-PPIL2 localied to DSBs induced in U2OS cells by laser microirradiation. EGFP-PPIL2 was expressed in U2OS cells. **(B)** PPIL2 enrichment at DSBs. ChIP assay was performed in ER-*Asi*SI U2OS cells were treated with 4-OHT (300 nM) for 4 h, using Flag M2 besds IP Flag-vector (CTR) or Flag-PPIL2. ChIP efficiencies were measured by qPCR from *Asi*SI induced DSBs. ER-*Asi*SI U2OS cells were treated with ATM inhibitor (ATMi) KU-55933 (20 μM, 1 h). **(C)** Endogenous interaction between PPIL2 and ZNF830. U2OS cells were collected and lysed using an anti-ZNF830 antibody for IP and western blotting using an anti-PPIL2 or anti-ZNF830 antibody. **(D)** Schematic diagram of full-lengthPPIL2 and truncations. **(E)** IP and Western blotting of recombinant GST-ZNF830 and Flag-PPIL2 constructs epressed in 293T cells. **(F)** GST pulldown and Western blot analysis using purified GST-ZNF830 and His-PPIL2 epressed in *E. coli*. CBB is coomassie brilliant blue. **(G)** Schematic diagram of ful-length ZNF830 and truncations. **(H)** GST pulldown and Western blot analysis using purified GST-ZNF830 and His-PPIL2 241–520 epressed in *E. coli*. CBB is coomassie brilliant blue. **(I)** PPIL2 enrichment of DSBs is regulated by ZNF830. ChIP assay was performed in ER-*Asi*SI U2OS cells treated with 4-OHT (300 nM) for 4 h, using Flag M2 besds IP Flag-vector (CTR) or Flag-PPIL2 and ER-*Asi*SI U2OS cells were infected with an shRNA control or shZNF830. The data represent the means of three independent experiments, with error bars as SEM and *p* values as noted: **p ≤ 0.01.

PPIL2 is an E3 ubiquitin ligase containing U-box domains (Hatakeyama et al., 2001). In many studies, PPIL2 is involved in the metastasis of cancer cells. The knockdown of PPIL2 led to a decrease in F-actin deposition, thereby influencing cell morphology and dynamics (Gaji et al., 2013). PPIL2 has been found to be a target of miR-31, which is related to cell invasion and migration (Henriksen et al., 2014). Recent studies have shown that PPIL2 affects the epithelial–mesenchymal transition (EMT) process through SNAl1 ubiquitination and degradation, which inhibits migration and invasion of breast cancer cells (Jia et al., 2017). Given that PPIL2 is involved in cancer metastasis, we sought to confirm whether PPIL2 is related to DNA repair. We first performed live-cell imaging to test whether PPIL2 is recruited to DSB sites through fluorescence monitoring. DSBs were induced in U2OS cells expressing EGFP-tagged PPIL2 using laser microirradiation. Live-cell fluorescence imaging showed the EGFP-PPIL2 accumulated in the laser-induced damage regions, indicating recruitment to the DBS sites (Figure 1A). At the same time, we added 4-hydroxytamoxifen (4-OHT) to U2OS cells which stably expressing the restriction enzyme *Asi*SI fused to a modified estrogen receptor ligand-binding domain. 4-OHT-induced *Asi*SI is able to generate multiple sequence-specific and unambiguously positioned DSBs throughout the genome. Chromatin immunoprecipitation (ChIP) analysis revealed that Flag-tagged PPIL2 was enriched at DSB sites and was regulated by ATM inhibitors (Figure 1B; Supplementary Figure S1A). Next, we found that neither the N-terminal nor the C-terminal construct of PPIL2 could be recruited to DNA damage sites. Thus, we think that the full length of PPIL2 is necessary for DNA damage recruitment (Supplementary Figure S1B), and that PPIL2 can be recruited to DSB sites in G1 phase as well as G2 phase (Supplementary Figure S1C). Additionally, PPIL2 was shown to decrease during the M phase in U2OS cells on synchronized cell cycles (Supplementary Figure S1D).

### PPIL2 interacts with ZNF830

ZNF830 was recently shown to participate in DNA damage repair through its interaction with CtIP (Chen et al., 2018). To confirmed this,a coimmunoprecipitation (co-IP) assay was performed to determine if ZNF830 also interacts with PPIL2 (Figure 1C). Full-length PPIL2 contains two functional domains: a U-box domain and a peptidylprolyl isomerase (PPIase) domain (Figure 1D) (Jia et al., 2018). To identify which fragment of PPIL2 is required for the binding of ZNF830, we generated Flag-tagged PPIL2 1–240 and Flag-tagged PPIL2 241–520. The IP assay revealed that PPIL2 241–520 interacted with GST-tagged ZNF830 (Figure 1E). Next, we used a GST-pulldown assay to probe the GST-tagged ZNF830 and full length or fragments of His-tagged PPIL2, which showed that ZNF830 interacted directly with PPIL2 241–520 (Figure 1F). In the CBB, we also found that PPIL2 241–520 can strongly interact with ZNF830 (Figure 1F right). We then narrowed down the fragment where PPIL2 interacts with ZNF830 and found that GST-tagged ZNF830 151–372 interacted with HIs-tagged PPIL2 241–520 (Figures 1G,H). We next performed ChIP to test whether PPIL2 was recruited to DSB sites in the absence of ZNF830. We observed that PPIL2 recruitment to DSB sites was reduced when ZNF830 expression was inhibited (Figure 1I; Supplementary Figure S1E). These results suggest that PPIL2 is recruited to DSB sites in a ZNF830-dependent manner.

### PPIL2 regulates CtIP-dependent HR

**FIGURE 2:**
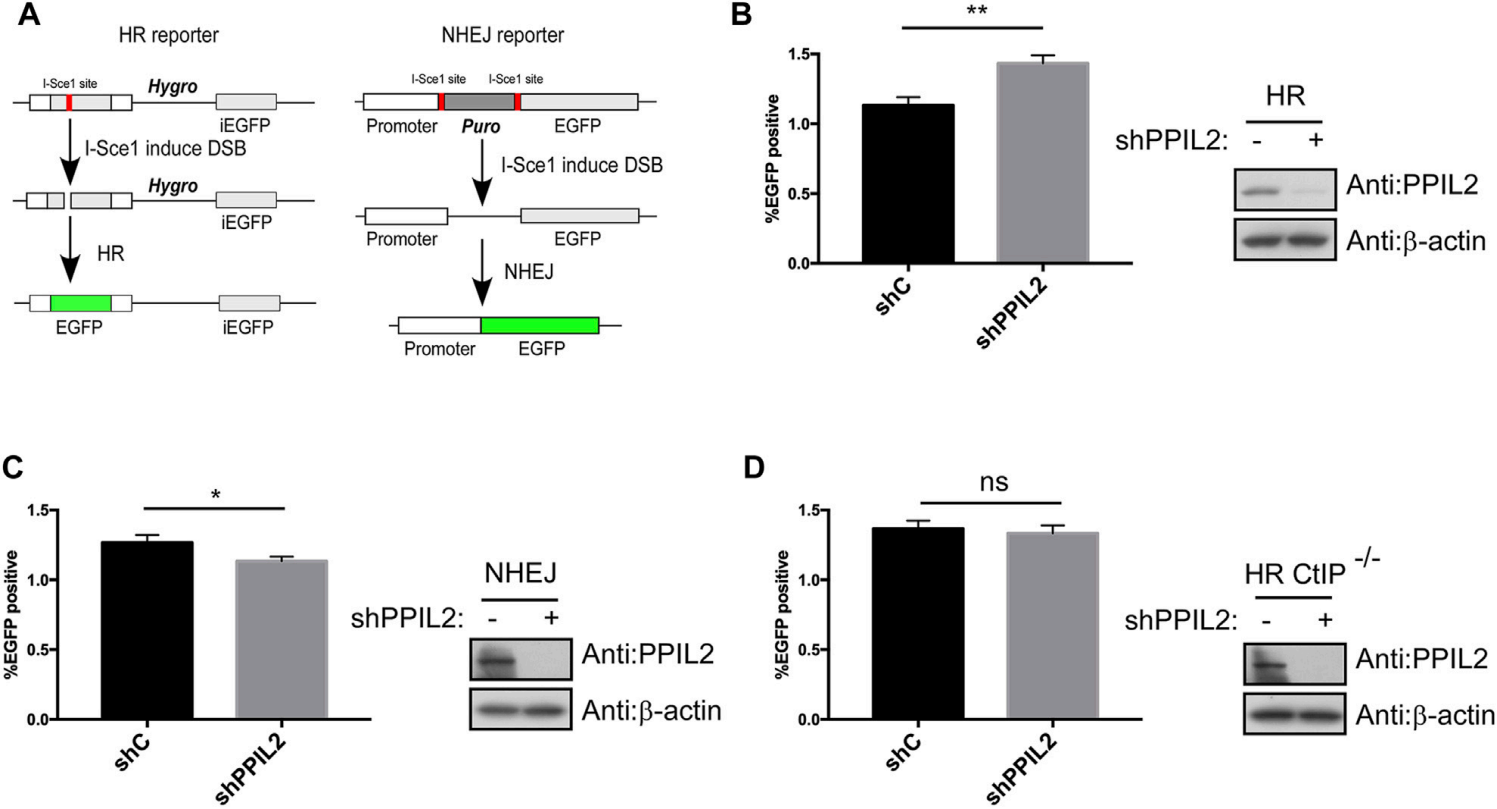
PPIL2 regulates HR in a CtIP-dependent manner. **(A)** Schematic of the EGFP-HR and EGFP-NHEJ reporter system. **(B,C)** Knockdown of PPIL2 promotes HR and inhibits NHEJ. EGFP-HR, and EGFP-NHEJ reporters were transfected into U2OS cells, and then were infected with shRNA control (shC) or shPPIL2, followed by infection with lentivirus-I-sceI for 16 h. After 48 h, the cells were collected, and GFP-positive cells were recorded by FACS. Left, the relative HR or NHEJ efficacy was quantified. Right, western blotting shows the efficiency of shPPIL2. **(D)** PPIL2 does not affect HR after CtIP depletion. EGFP-HR was performed in U2OS CtIP-depleted cells using the same method as the EGFP-HR reporter. Top, the relative HR efficacy was quantified. Bottom, western blotting shows the efficiency of shPPIL2. The data represent the means of three independent experiments, with error bars as SEM and *p* values as noted: *p ≤ 0.05; **p ≤ 0.01; n. s not significant.

After finding that PPIL2 is recruited to DNA damage sites, we used a I-SceI-mediated reporter system to measure whether PPIL2 affects HR, NHEJ and MMEJ activity (Figure 2A; Supplementary Figure S2A) (Wang et al., 2012). The percentage of GFP-positive cells increased in cultures treated with PPIL2 shRNA, indicating PPIL2-dependent suppression of the HR repair pathway (Figure 2B). Conversely, using the PPIL2 shRNA infection reporter system to detect NHEJ and MMEJ activity, we observed that PPIL2 reduced NHEJ and MMEJ efficiency when PPIL2 was knocked down (Figure 2C; Supplementary Figure S2B). Since CtIP is a key factor in the HR pathway (Sartori et al., 2007), we investigate the relationship between PPIL2 and CtIP by suppressing PPIL2 expression in CtIP-depleted EGFP-HR reporter cells. Knocking down PPIL2 did not further reduce HR repair (Figure 2D), indicating that PPIL2 is downstream of CtIP in the HR pathway. We next performed live-cell imaging to test whether PPIL2 is recruited to DSB sites by monitoring fluorescence without CtIP. We observed that EGFP-tagged PPIL2 accumulated in the laser-induced damage region and was independent of CtIP (Supplementary Figure S2C). These results suggest that PPIL2 inhibits HR in a CtIP-dependent manner.

### PPIL2 interacts with CtIP

**FIGURE 3:**
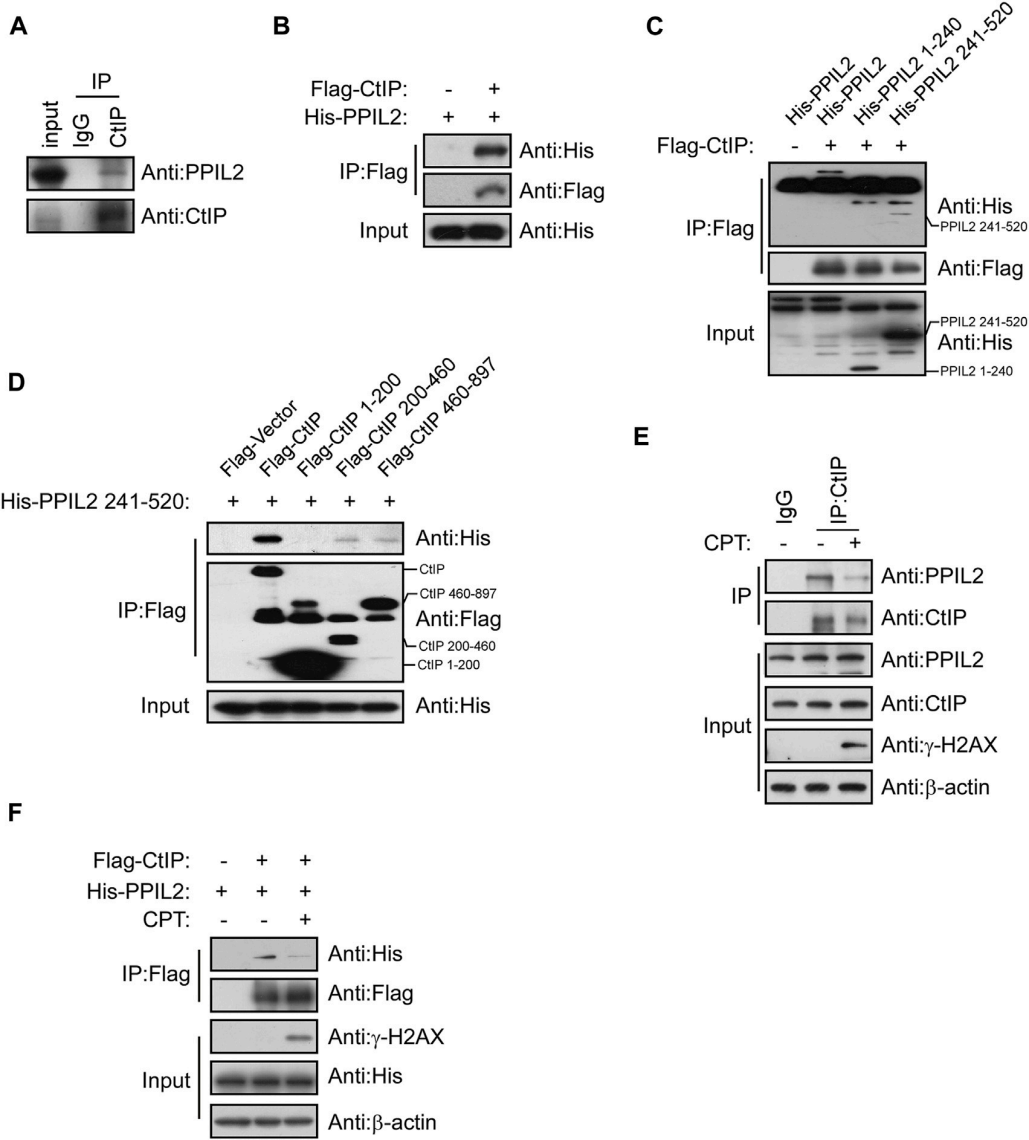
PPIL2 interacts with CtIP. **(A)** Western blotting of U2OS cells lysed in the presence of anti-CtIP antibody with anti-CtIP and anti-PPIL2 antibodies show an endogenous interaction between PPIL2 and CtIP. **(B)** 293T cells were cotransfected for recombinant Flag-CtIP and His-PLK1 expression. IP with anti-Flag and western blotting with anti-His and anti-Flag. **(C)** 293T cells were cotransfected with His-PPIL2 fragments and Flag-CtIP, followed by IP with Flag and western blot analysis. **(D)** 293T cells were cotransfected with Flag-CtIP fragments and His-PPIL2 241–520, followed by IP with Flag and western blot analysis. **(E)** Co-IP assay probing PPIL2-CtIP interaction in U2OS cells treated with 2 μM CPT for 1 h **(F)** 293T cells were cotransfected with Flag-CtIP and His-PPIL2 and treated with or without 2 μM CPT for 1 h. The association between PPIL2 and CtIP was analysed by IP with Flag and western blot.

A co-IP assay was performed to determine if PPIL2’s CtIP-dependent regulation of HR is mediated by interaction between the two. Co-IP showed that CtIP interaction with PPIL2 (Figures 3A,B). To identify the region(s) of PPIL2 required for CtIP binding, we generated His-tagged truncations of PPIL2 residues 1–240 and 241–520. The IP assay showed that PPIL2 241–520 interacted with Flag-tagged CtIP (Figure 3C). Moreover, PPIL2 241–520 interacted with Flag-tagged CtIP 200–460 and 460–897 (Figure 3D). These data indicate that the PPIL2 C-terminus, interacts with CtIP C-terminus. To confirm if the interaction between CtIP and PPIL2 is affected by DNA damage, we tested endogenous IP and co-IP after treatment with 2 μM camptothecin (CPT). We found that the interaction between CtIP and PPIL2 decreased when canptothecin-induced DSBs occurred (Figures 3E,F). These results indicate that CtIP interacts with PPIL2 and that this association is suppressed by DNA damage.

### Ubiquitination of CtIP by PPIL2

**FIGURE 4:**
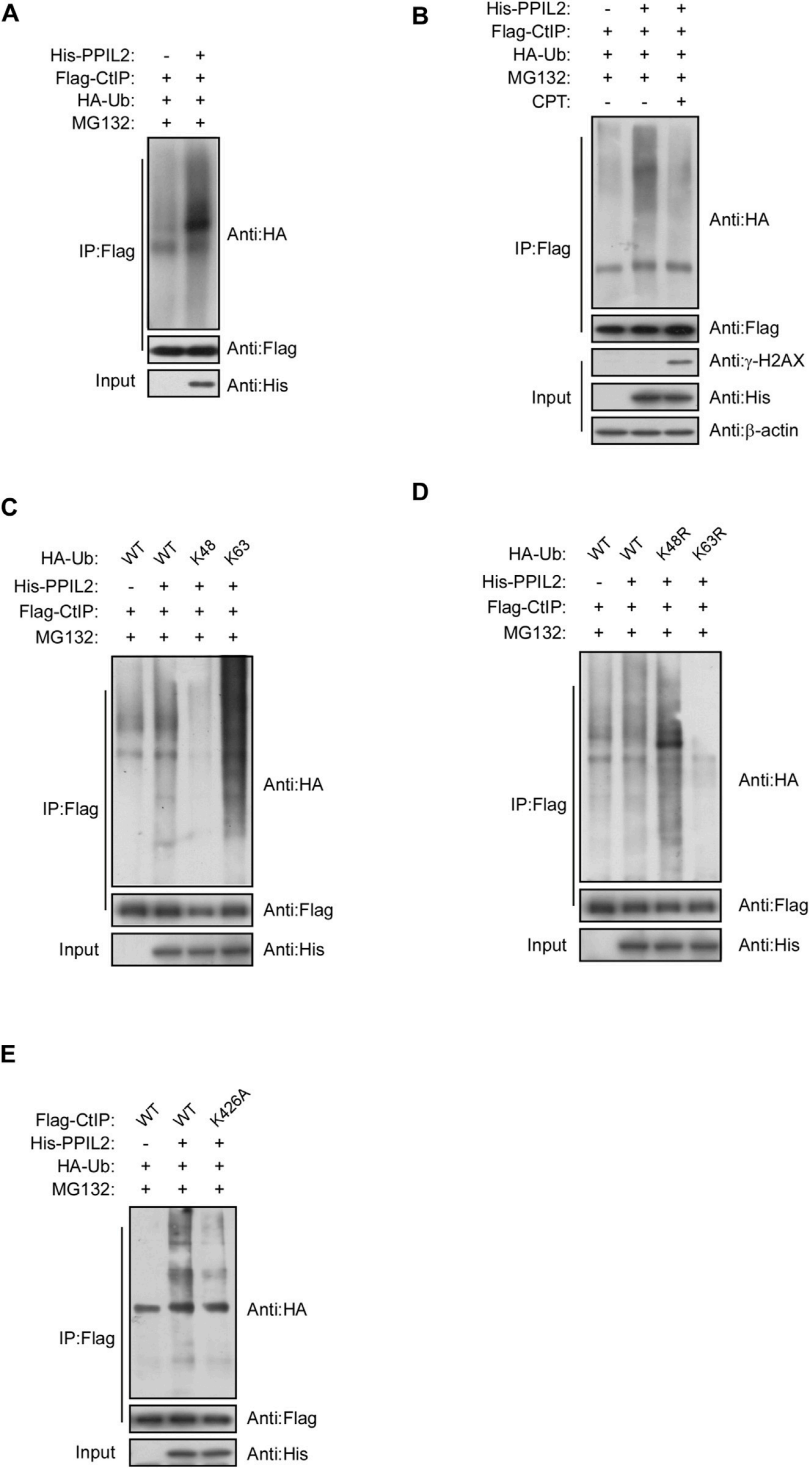
PPIL2 ubiquitination of CtIP characterized by co-IP. **(A)** 293T cells were cotransfected with Flag-CtIP, His-PPIL2, and HA-Ub. 48 h after transfection, the cells were treated with MG132 (20 μM) for 3 h. The cells were lysed in NETN buffer containing a protease inhibitor cocktail (PIC), and the cell lysate was immunoprecipitated with anti-Flag and analysed by immunoblotting with anti-HA. This process was repeated on **(B)** cells treated for 1 h with 2 μM CPT; **(C)** cells cotransfected with Flag-CtIP, His-PPIL2 and HA-Ub WT, K48 or K63; **(D)** cells cotransfected with Flag-CtIP, His-PPIL2 and HA-Ub WT, K48R or K63R; and **(E)** cells cotransfected with His-PPIL2, HA-Ub and Flag-CtIP WT or K426A mutant.

We investigated the role of PPIL2, a U-box-type E3 ligase in regulating the ubiquitination of CtIP, and observed that PPIL2 increases CtIP ubiquitination (Figure 4A). As expected, PPIL2 ubiquitination of CtIP decreased in cells with DSBs (Figure 4B). When DSBs occur, the interaction between PPIL2 and CtIP becomes less stable (Figures 3E,F), and ubiquitination is subsequently inhibited. To verify the ubiquitin linkage type of PPIL2 ubiquitination of CtIP, we prepared cells transfected with HA-Ub K48 and HA-Ub K63, ubiquitin plasmids with all ubiquitination site lysines mutated except for K48 and K63, respectively, to identify the type of CtIP ubiquitination catalyzed by PPIL2. K63-linked polyubiquitylation of CtIP increased in the presence of overexpressed PPIL2, while K48-linked polyubiquitylation of CtIP decreased (Figures 4C,D). To determine which CtIP residues PPIL2 ubiquitinates, we cotransfected 293T cells with Flag-CtIP, His-PPIL2 and HA-Ub, followed by IP Flag, and then identified the ubiquitinated sites *via* mass spectrometry. We found that CtIP is ubiquitinated by PPIL2 at K426 (Figure 4E; Supplementary Table S1). These results indicate that CtIP is a ubiquitination substrate of PPIL2, which ubiquitinates the CtIP K426 residue.

### PLK1 interacts with and phosphorylates PPIL2

**FIGURE 5:**
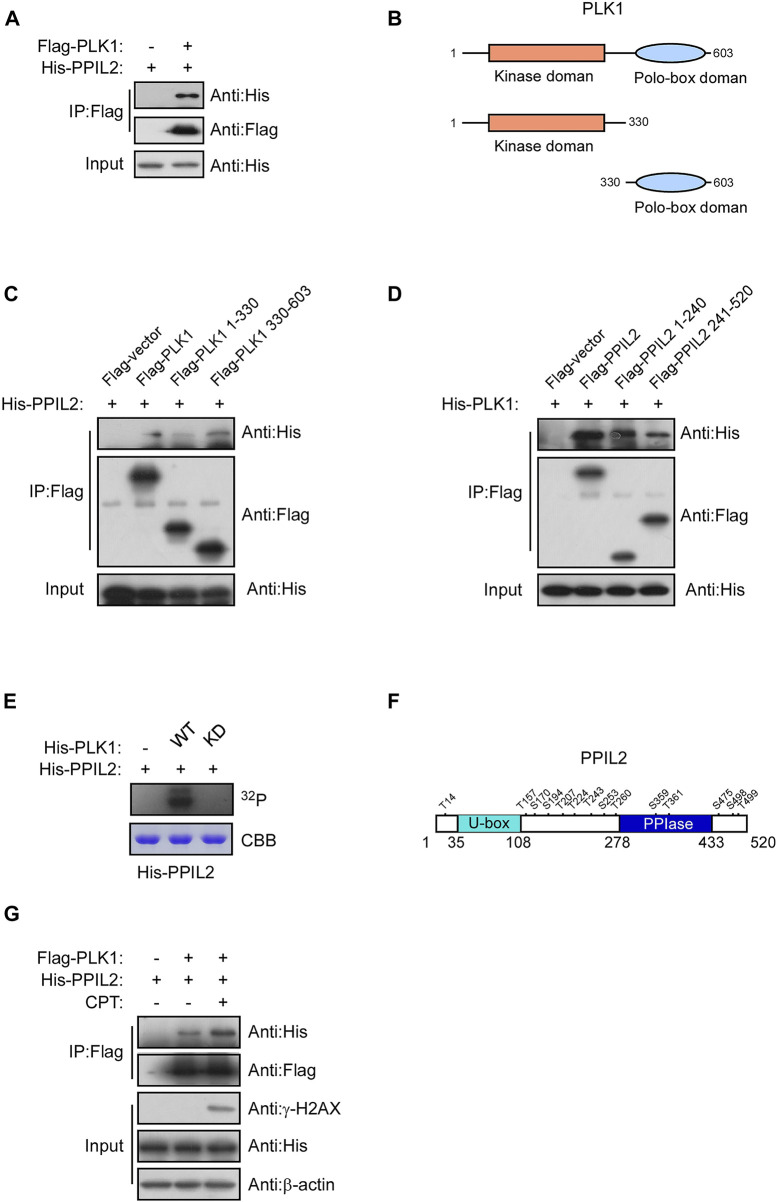
PLK1 interacts with and phosphorylates PPIL2. **(A)** 293T cells were cotransfected with Flag-PLK1, and His-PPIL2 expression constructs. IP and western blotting were performed with the indicated antibodies. **(B)** Schematic diagram of various PLK1 fragments. **(C)** 293T cells were cotransfected with Flag-PLK1 fragments and His-PPIL2, followed by IP with Flag and analysis by western blot. **(D)** 293T cells were cotransfected with Flag-PPIL2 fragments (see Figure 1C) and His-PLK1, followed by IP with Flag and western blot analysis. **(E)** Purified His-PPIL2 was incubated with [γ -^32^P] ATP in the presence of PLK1 WT or PLK1 KD for the *in vitro* kinase assay. The radiolabelled proteins were visualized following SDS–PAGE. Coomassie Blue staining indicates His-PPIL2 loading. **(F)** PLK1 phosphorylation of PPIL2 was analysed by mass spectrometry, and the potential PLK1 target sites on PPIL2 were identified. **(G)** 293T cells were cotransfected with Flag-PLK1 and His-PPIL2 and treated with or without 2 μM CPT for 1 h. The association between PPIL2 and PLK1 was analysed by IP with Flag and western blot.

Subsquent to our finding that PPIL2 is associated with PLK1 (Figure 5A), we use immunoprecipitation of different PPIL2 and PLK1 truncations to determine what regions are involved directly in their interaction. We observed an interaction between PLK1 residues 330–603, the polo-box domain (PBD) and full length of the PPIL2 (Figures 5B–D). Either the N-terminal domain or C-terminal domain of PPIL2 can interact with PLK1, and PPIL2 mainly interacts with the PBD domain of PLK1. The PBD domain has two polo-box structures and connected by a flexible linker. The crystal structure of PLK1 shows that the two polo-box domain are similar and can interact with substrate (Liu et al., 2017). A complete crystal structure of PPIL2 has not been reported. Acording to the structure prediction of AlphaFoldDB (Jumper et al., 2021), the N-terminus and C-terminus of PPIL2 are located on the same side (Supplementary Figure S3A), hence it is reasonable that the PLK1 PBD domain is able to interact with both the N- and C-terminus of PPIL2.

Given that PPIL2 binds to the PLK1, we tested whether PPIL2 is a subtstrate for PLK1 serine/threonine phosphorylation. An *in vitro* kinase assay showed that PLK1 can phosphorylate PPIL2 (Figure 5E). We performed another *in vitro* kinase assay followed by mass spectrometry to determine the PPIL2 residues phosphorylated by PLK1, and identified 14 potential phosphorylation sites (Figure 5F; Supplementary Table S2). We also tested the interaction between PPIL2 and PLK1 under DNA damage stress. We detected that the interaction between PPIL2 and PLK1 was increased when the DSBs occured (Figure 5G). These results indicate that PPIL2 interacts with PLK1 and can be phosphorylated by PLK1.

Curiously, we did not find evidence that PPIL2 ubiquitinates PLK1. In fact, PPIL2 appears to protect it from ubiquitination by endogenous ligases (Supplementary Figures S3B,C), and this protection is affected by the activity of PLK1 and the phosphorylation of PPIL2 by PLK1 (Supplementary Figures S3D,E), suggesting a different regulatory relationship from direct ubiquitination (Supplementary Figure S3).

### Phosphorylation of PPIL2 by PLK1 suppresses CtIP ubiquitination

**FIGURE 6:**
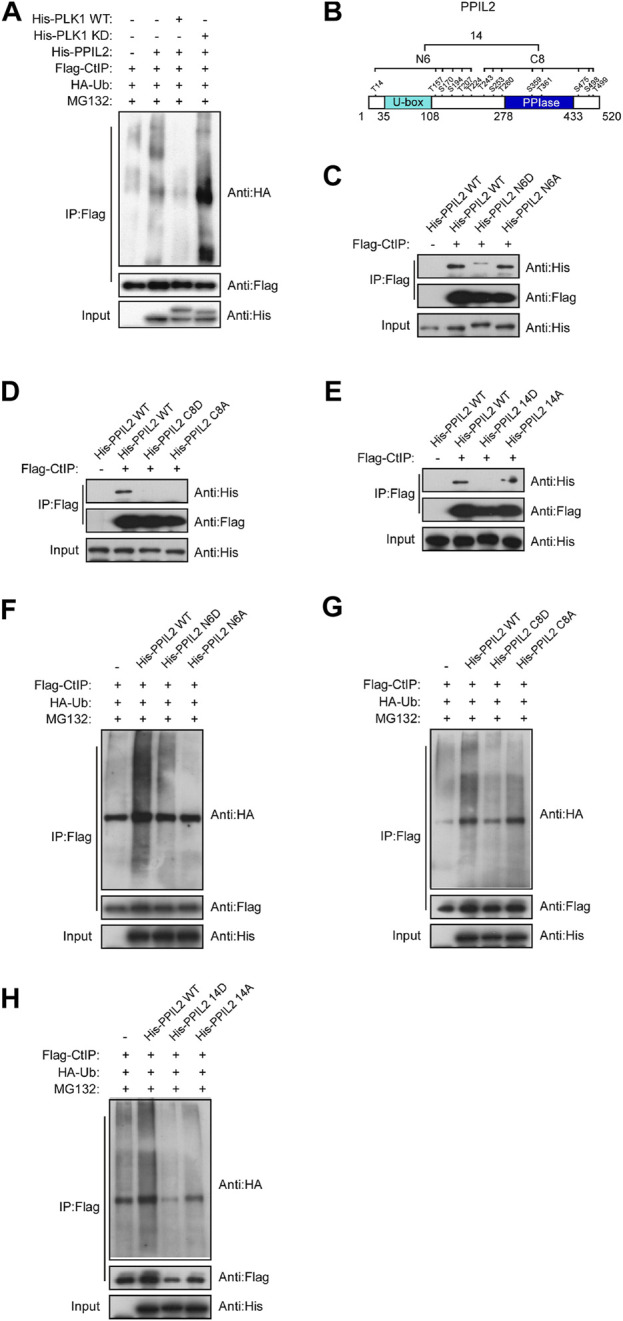
Phosphorylation of PPIL2 by PLK1 suppresses CtIP ubiquitination. **(A)** 293T cells were cotransfected with Flag-CtIP, His-PPIL2, HA-Ub, and His-PLK1 WT or kinase dead (KD). 48 h post transfection, the cells were treated with MG132 (20 μM) for 3 h. The cells were then lysed in NETN buffer containing a protease inhibitor cocktail (PIC), and the cell lysate was immunoprecipitated with anti-Flag and analysed by immunoblotting with anti-HA. **(B)** Schematic diagram of the potential PLK1 phosphorylation target site on PPIL2. **(C,D,E)** 293T cells were cotransfected with Flag-CtIP and His-PPIL2 mutant expression constructs and then assayed as in **(A) (F,G,H)** 293T cells were cotransfected with Flag-CtIP, HA-Ub, and His-PPIL2 WT or His-PPIL2 mutants, and then assayed as in **(A)**

Given that PPIL2 ubiquitinates CtIP (Figure 4A) and PLK1 phosphorylates PPIL2 (Figure 5E), we hypothesized that phosphorylation of PPIL2 by PLK1 may affect ubiquitination of CtIP by PPIL2. To investigate this, we overexpressed WT and PLK1 mutated to be kinase-dead (KD). We found that WT PLK1 inhibited PPIL2 ubiquitination of CtIP, but not KD PLK1 (Figure 6A). PLK1 can phosphorylate PPIL2 at 14 residues (Figure 5F), six flanking the U-box domain (residues 1–240) and eight on and adjacent to the PPIase domain (residues 241–520). Hence, we divided these 14 sites into two fragments, N6 and C8 (Figure 6B), and generated mutants replacing the serine/threonine phosphorylation sites of them and the full-length PPIL2 with asparate (N6D, C8D, and 14D) and alanine (N6A, C8A, and 14A). IP showed that the interaction with CtIP was weaker for all of the mutant constructs (Figures 6C–E). Partial single-point mutations of full-length PPIL2 produced similar results to the same as multi-point mutations, showing reduced binding to CtIP (Supplementary Figure S4).

Mutation of the PPIL2 phosphorylation sites also reduced CtIP ubiquitination. CtIP was ubiquitinated less in the presence of PPIL2 D and A mutants compared to WT PPIL2 (Figures 6F–H). This was further supported by partial single-point mutations, as shown in Supplementary Figure S5. These data indicate that PLK1 phosphorylates PPIL2, which in turn inhibits PPIL2 ubiquitination of CtIP.

### Ubiquitination of CtIP by PPIL2 suppresses HR and DSB end resection

**FIGURE 7:**
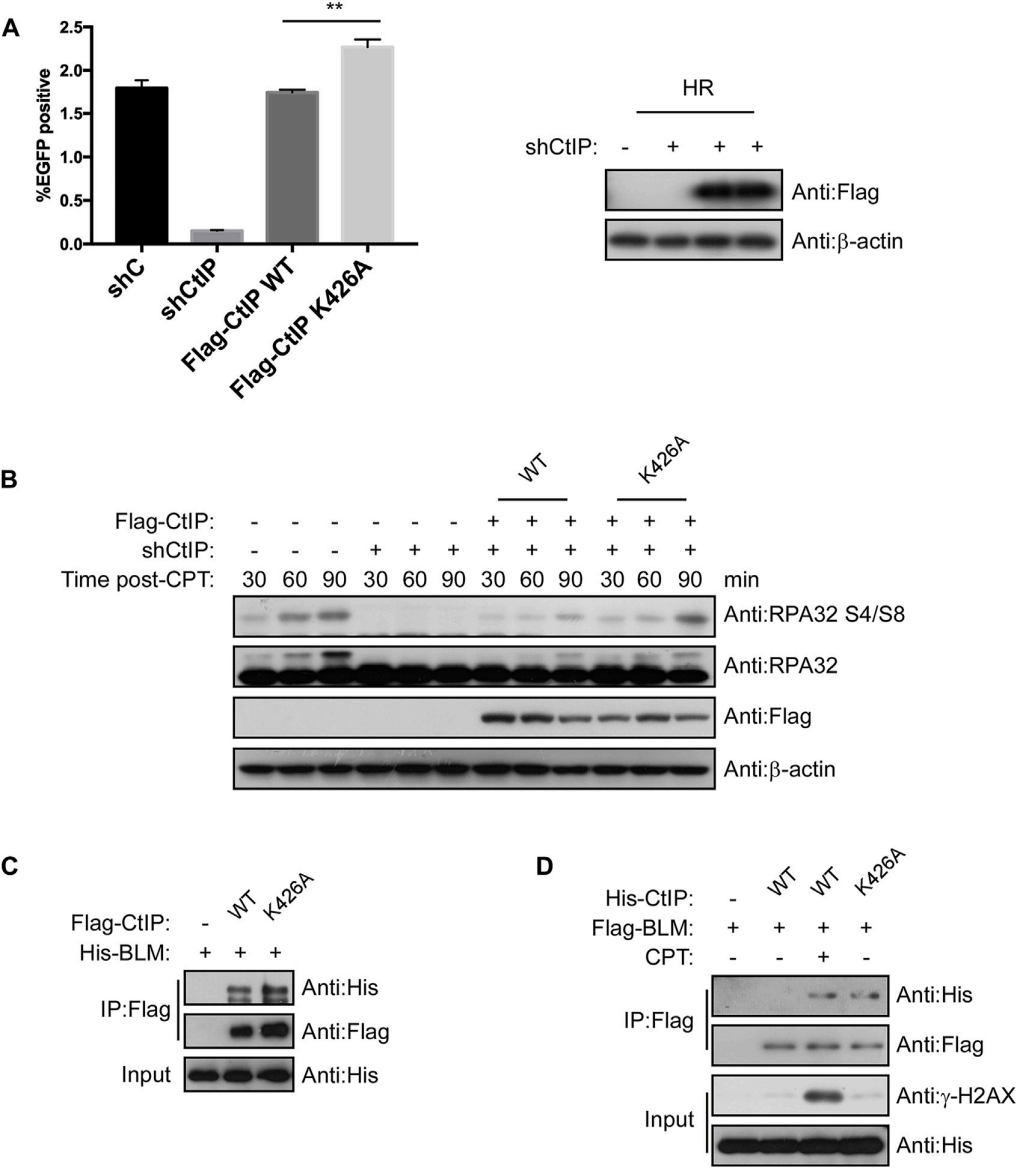
Ubiquitination of CtIP by PPIL2 suppresses HR and DSB end resection. **(A)** An EGFP-HR assay was performed in U2OS cells stably expressing Flag-CtIP WT or K424A mutant, and U2OS cells were infected with an shRNA control (shC) or shPPIL2. Western blotting shows the expression of Flag-CtIP variants. **(B)** U2OS cells were transfected to stably express Flag-CtIP WT or K426A mutants and treated with shCtiP to silence endogenous CtIP expression, treated with CPT (2 μM) for 1 h and released at different times, and then assayed *via* Western blotting with the indicated antibodies. **(C)** IP and Western blotting with the indicated antibodies of lysate from 293T cells were cotransfected with His-BLM and Flag-CtIP mutant expression constructs. **(D)** 293T cells were cotransfected with Flag-BLM and His-CtIP mutant expression constructs. Cells were treated with CPT (2 μM) for 1 h. IP and western blotting with the indicated antibodies. The data represent the means of three independent experiments, with error bars as SEM and *p* values as noted: **p < 0.01.

Since PPIL2 ubiquitinates CtIP at K426, we next tested whether this modification promotes CtIP function. We employed an HR reporter systems expressing WT CtIP or the K426A mutant. We found that HR was more efficiative, with the K426A mutant with WT (Figure 7A) but NHEJ and MMEJ were not (Supplementary Figure S6). Cells treated with 2 μM CPT for 1 h showed activation of S4/S8 RPA2 phosphorylation, a marker of DNA end resection (Nimonkar et al., 2011), within 30–90 min, but not in cells treated with shCtIP (Figure 7B). Re-expression of WT and K426A CtIP fully rescued end-resection. Additionally, RPA2 S4/S8 phosphorylation in CtIP K426A cells was higher than in CtIP WT cells (Figure 7B). DNA helicase BLM, a key factor in long-range resection, is known to interact with CtIP to promote HR (Daley et al., 2017). We conducted a co-IP assay to verify whether CtIP K426A mutation affects BLM-dependent DNA resection, (Figures 7C,D). We found that CtIP K426A enhanced the interaction with BLM. As the mutant can not be ubiquitinated by PPIL2, thus these results indicate that PPIL2 ubiquitination of the CtIP negative control HR and DSB end resection.

## Discussion

The DNA damage response (DDR) is essential to maintianing genome stability in response to constant exogenous and endogenous damage. Ubiquitin-mediated posttranslational modifications play an important role in maintaining genome stability by orchestrating key DDR events, including various DNA repair pathways (Jackson and Durocher 2013). We found that PPIL2 can inhibit HR, but does not have much effect on NHEJ and MMEJ, and this inhibition depends on CtIP. When we silenced the expression of PPIL2, the efficiency of HR repair was promoted. In CtIP-deficient cells, inhibition of PPIL2 did not affect the efficiency of HR repair, showing that PPIL2 is downstream of CtIP in the HR pathway. CtIP has repeatedly been shown to be required for all types of homology-directed repair mechanisms, including HR and MMEJ. In our study, we found CtIP K426A increasing HR but decreasing MMEJ compared to CtIP WT (Figure 7A; Supplementary Figure S6B). In response to this phenomenon, we think that the CtIP K426A mutant is more inclined to promote HR repair, because K426A can promote the interaction of CtIP with the long-range resection factor BLM during HR repair.

E3 ubiquitin ligase has been known to interact with CtIP and may affect DNA end resection. In our study, we found that PPIL2 interacts with CtIP and ubiquitinates CtIP at the K426 site. Prior researchs demonstrated that E3 ubiquitin ligase interact with CtIP and ubiquitination of CtIP promotes the activity of CtIP, such as the ring-type E3 ligase BRCA1 BRCT domain ubiquitinates CtIP and promotes CtIP recruitment to DNA damage sites (Yu, Fu, Lai, Ba Er and Chen 2006). In addition, the RNF138 and E2-binding enzyme UBE2D complex interacts with CtIP to promote CtIP ubiquitination and accumulation at DSB sites (Schmidt et al., 2015). This shows that the ubiquitination of CtIP by RNF138-UBE2D is a key step in promoting HR. The author identified 13 ubiquitinated lysines in CtIP by mass spectrometry, excluding the K426 site. Unlike the above studies, we found ubiquitination of CtIP by PPIL2 inhibits DNA end resection and HR repair of CtIP. When DSBs occurred, the interaction between PPIL2 and CtIP was decreased, and the ubiquitination of CtIP was weakened, thereby restoring the DNA end resection activity of CtIP.

**FIGURE 8:**
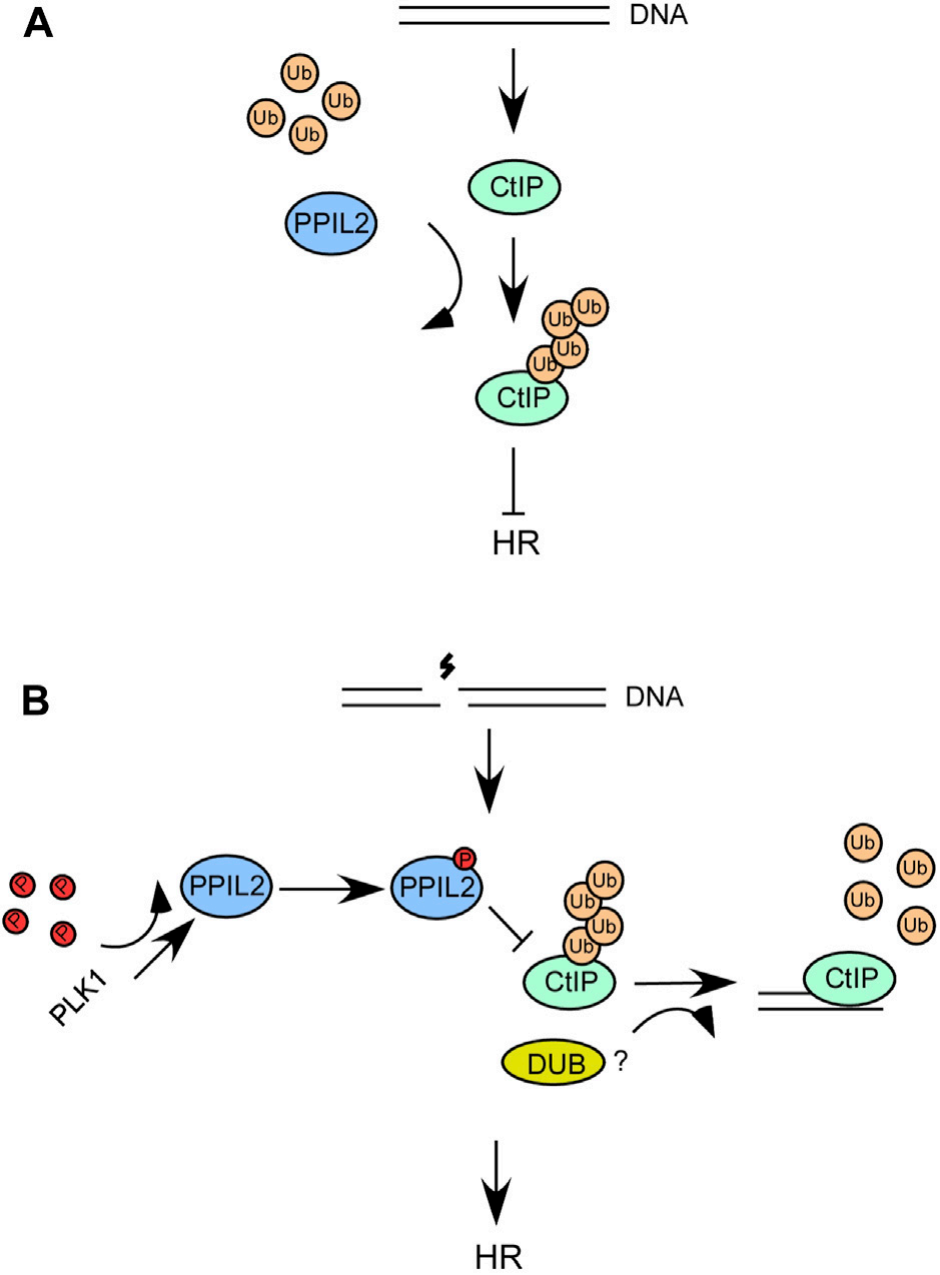
**(A,B)** A schematic describing the role of PPIL2 ubiquitination of CtIP and how it affects HR and DSB end resection.

Interestingly, two prior studies demonstrated that the deubiquitinating enzyme USP4 plays a role in DNA end resection (Liu et al., 2015; Wijnhoven et al., 2015). USP4 interacts with CtIP and MRN and promotes CtIP recruitment and DNA repair. As such, ubiquitination and deubiquitination can affect the DNA end resection function of CtIP. In a recent report, USP52 was revealed to interact with CtIP and deubiquitinate it, thereby promoting DNA end resection and HR (Gao et al., 2020). Over the course of this study, we also sought to verify whether USP52 or USP4 deubiquitinate the CtIP K426 site. However, we have not observed the deubiquination of CtIP K426 by USP52 and USP4 (Supplementary Figure S7). It is possible another DUBs involved in this process (Figure 8B).

PLK1 expression peaks in the G2/M phase and its role in mitosis is well characterized (Golsteyn et al., 1995), but relatively little is known about its function in S phase. Prior studies have shown that PLK1 accumulates in the nucleus during S phase, during which it PLK1 phosphorylates and activates topoisomerase II (Li et al., 2008) and that PLK1 stimulates S2/G2 phase DNA repair function by phosphorylating Rad51 (Yata et al., 2012). PLK1 is enriched at DSB sites within seconds in a PARP-1-dependent manner. Poly (ADP-) ribose (PAR) chains directly bind to PLK1 and inhibit its enzymatic activity. CHK1-PLK1-RAD51 axis ultimately promotes HR-mediated repair (Peng et al., 2021) (Li et al., 2019). In our study, we identified PPIL2 as another S/G2 phase substrate of PLK1. We determined full length PPIL2 interacts with PLK1 PBD domain and PLK1 phosphorylates PPIL2 at 14 sites. This interaction increase in the present of DSBs, and inhibition of CtIP ubiquitination further increases the activity of CtIP and promotes HR. PPIL2 also can help stabilize PLK1 through the phosphorylation activity of PLK1 and the 14 sites of PPIL2 phosphorylated by PLK1, and may further promote the accumulation of PLK1 and increase the activity of PLK1.

In this study, we found that PPIL2 interacts with CtIP to inhibit HR. We also found that ZNF830, which can bind to double-stranded DNA through its ZNF domain and is involved in HR. In our study, we found that ZNF830 can strongly interact with PPIL2 (Figure 1F) and help PPIL2 be recruited to DNA damage sites, and CtIP does not seems to affect the recruitment of PPIL2 (Supplementary Figure S2C). It remains unknown whether these interactions are concurrent, and if PPIL2 also ubiquitinates ZNF830. Our next step in this research will be to investigate the regulatory interactions between these three proteins and its affect on HR.

We propose a model wherein when DSBs occur, the interaction between PLK1 and PPIL2 increases, allowing PLK1 to phosphorylate PPIL2 (Figures 8A,B). Subsequently, PPIL2 ubiquitination of CtIP is weakened, and some DUBs may participate in this reaction. Therefore, the phosphorylation of PPIL2 by PLK1 inhibits the ubiquitination of CtIP by PPIL2, increasing the activity of CtIP and promoting HR. In addition, CtIP, which is not affected by PPIL2, can more effectively bind HR-related downstream proteins, such as BLM, which can further promote the HR process. Although it has been reported that the CtIP region between 161 and 369 is involved in BLM stimulation (Daley et al., 2017), it is possible that ubiquitination at the K426 site of CtIP inhibits the interaction of CtIP with BLM in the structural. Further research is needed to gain a better understanding of this system.

## Data Availability

The raw data supporting the conclusion of this article will be made available by the authors, without undue reservation.
