# Supplementary material for: PLK1-mediated phosphorylation of PPIL2 regulates HR via CtIP
Source: Front Cell Dev Biol. 2022 Aug 25;10:902403. doi: 10.3389/fcell.2022.902403 (PMC9452783; doi:10.3389/fcell.2022.902403)
Supplement: Supplementary file 3 [file Table1.DOCX]

**Supplementary Figure S1**


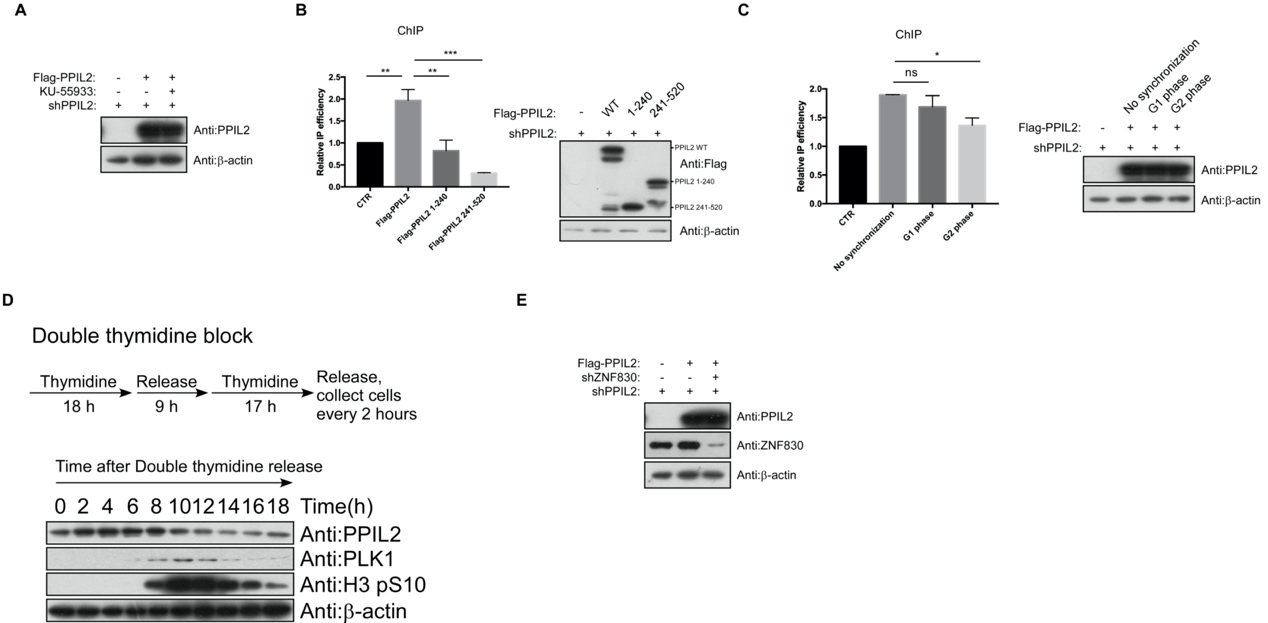


**Supplementary Figure S1.** (**A**) Western blotting shows the expression of Flag-PPIL2. (**B**) ChIP assay was performed in ER-*Asi*SI U2OS cells treated with 4-OHT (300 nM) for 4 h, using Flag M2 besds IP Flag-vector (CTR), Flag-PPIL2 full-length or truncations. ChIP efficiencies were measured by qPCR from *Asi*SI induced DSBs. (**C**) ChIP assay was performed in ER-*Asi*SI U2OS cells were treated with 4-OHT (300 nM) for 4 h, using Flag M2 beads IP Flag-vector (CTR) and Flag-PPIL2. ER-*Asi*SI U2OS cells were synchronized at G1 phase by double-thymidine block and then released for 10 h to G2 phase, followed by western blotting which shows the expression of Flag-PPIL2. (**D**) Up: Schematic of cell cycle synchronization strategy. Down: U2OS cells were synchronized at G1 phase by double-thymidine block and then released for 0, 4, 6, 8, 10,12,14,16,18 h, followed by western blotting with indicated antibody. (**E**) Western blotting shows the expression of Flag-PPIL2 and the inhibition efficiency of shZNF830. The data represent the means of three independent experiments, with error bars as SEM and P values as noted: *P ≤ 0.05; **P ≤ 0.01; ***P ≤ 0.001; n.s. not significant.

**Supplementary Figure S2.**

**
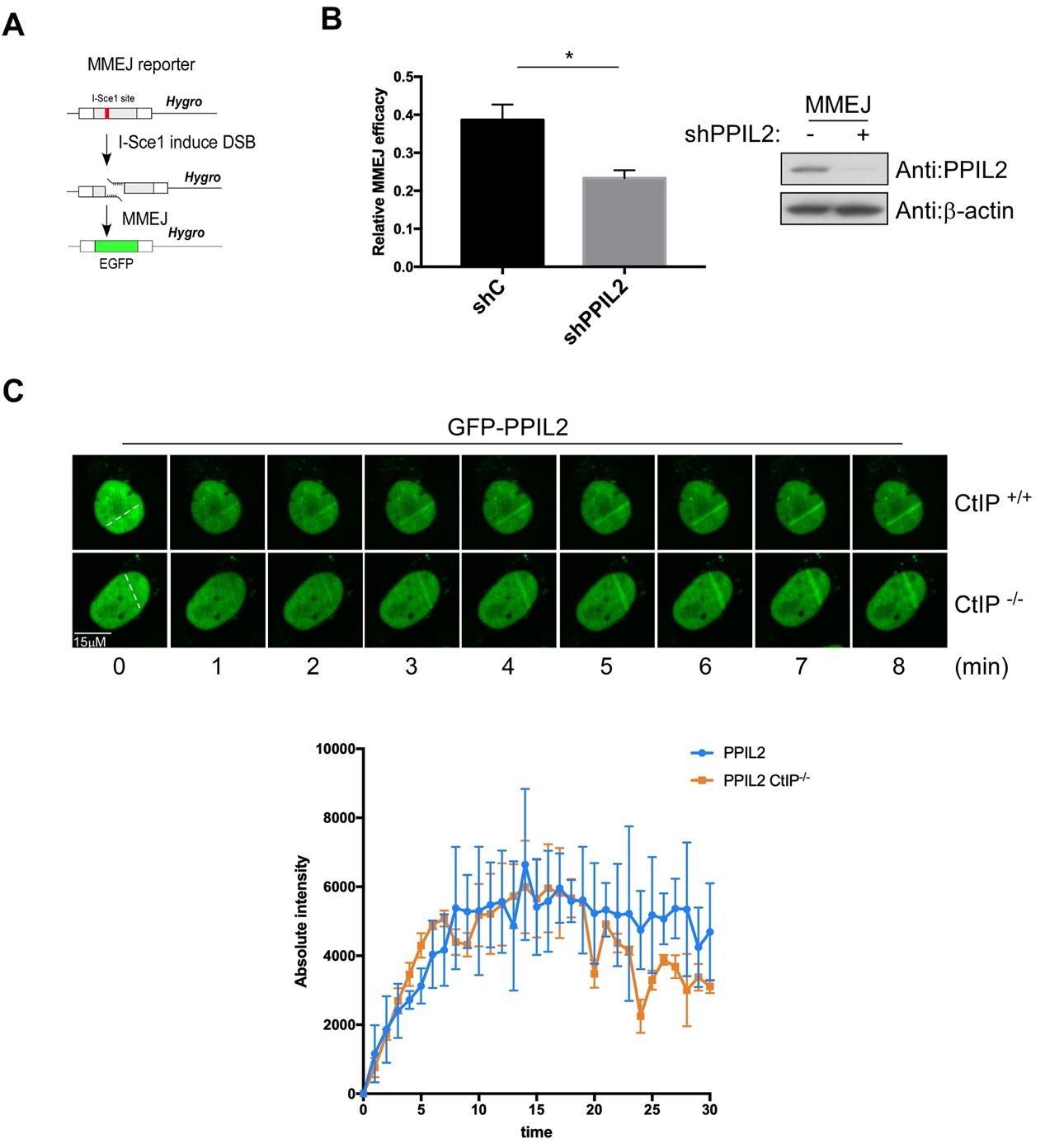
**

**Supplementary Figure S2.** Knockdown of PPIL2 inhibits MMEJ. (**A**) Schematic of the EGFP-MMEJ reporter system. (**B**) The EGFP-MMEJ reporter was transfected into U2OS cells which were then infected with shRNA control (shC) or shPPIL2 using the same method as the EGFP-HR reporter. (**C**) Recruitment of GFP-PPIL2 to DSBs was monitored in U2OS cells, in which with or without endogenous CtIP. The absolute intensity of the GFP-PPIL2 fluorescent signal was determined. The data represent the means of three independent experiments, with error bars as SEM and P values as noted: **p*<0.05.

**Supplementary Figure S3.**


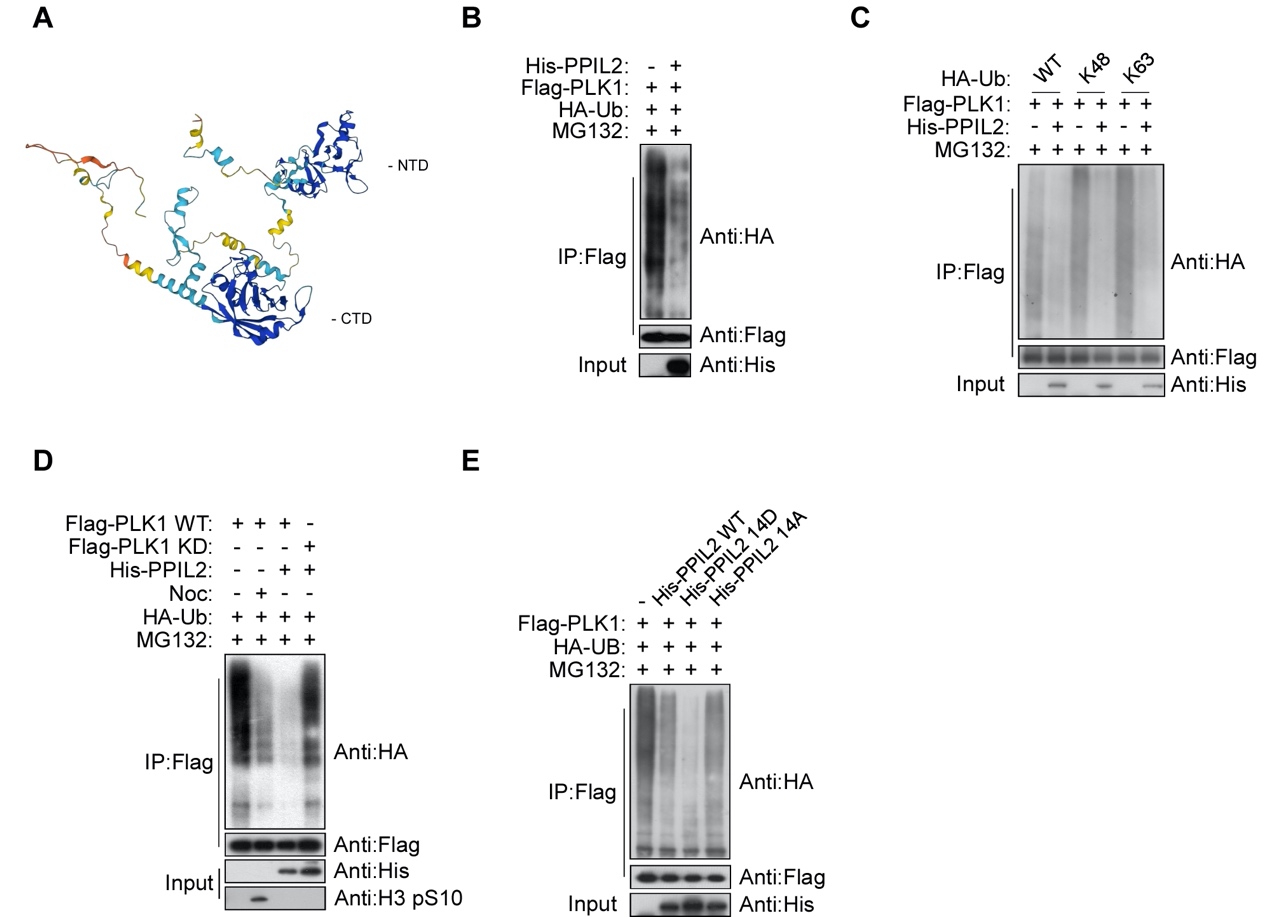


**Supplementary Figure S3.** PPIL2 inhibits ubiquitination of PLK1. (**A**) AlphaFoldDB structure prediction of PPIL2. (**B**) 293T cells were cotransfected with Flag-PLK1, His-PPIL2 and HA-Ub. 48 h post transfection, the cells were treated with MG132 (20 μM) for 3 h, then immunoprecipitated with anti-Flag and analysed by immunoblotting with anti-HA. (**C**) 293T cells were cotransfected with Flag-PLK1, His-PPIL2 and HA-Ub WT, K48 or K63, then immunoprecipitated with anti-Flag and analysed by immunoblotting with anti-HA. (**D**) 293T cells were cotransfected with Flag-PLK1 WT or KD, and His-PPIL2 and HA-Ub. 293T cells were cotransfected with Flag-PLK1 WT, His-PPIL2 and HA-Ub, and then treated with 340 nM nocodazole (Noc) for 16 h before harvesting. 48 h post-transfection, the cells were treated with MG132 (20 μM) for 3 h, then immunoprecipitated with anti-Flag and analysed by immunoblotting with anti-HA. (**E**) 293T cells were cotransfected with Flag-PLK1, HA-Ub and His-PPIL2 WT, 14D or 14A, 48 h post transfection, the cells were treated with MG132 (20 μM) for 3 h, then immunoprecipitated with anti-Flag and analysed by immunoblotting with anti-HA.

**Supplementary Figure S4.**


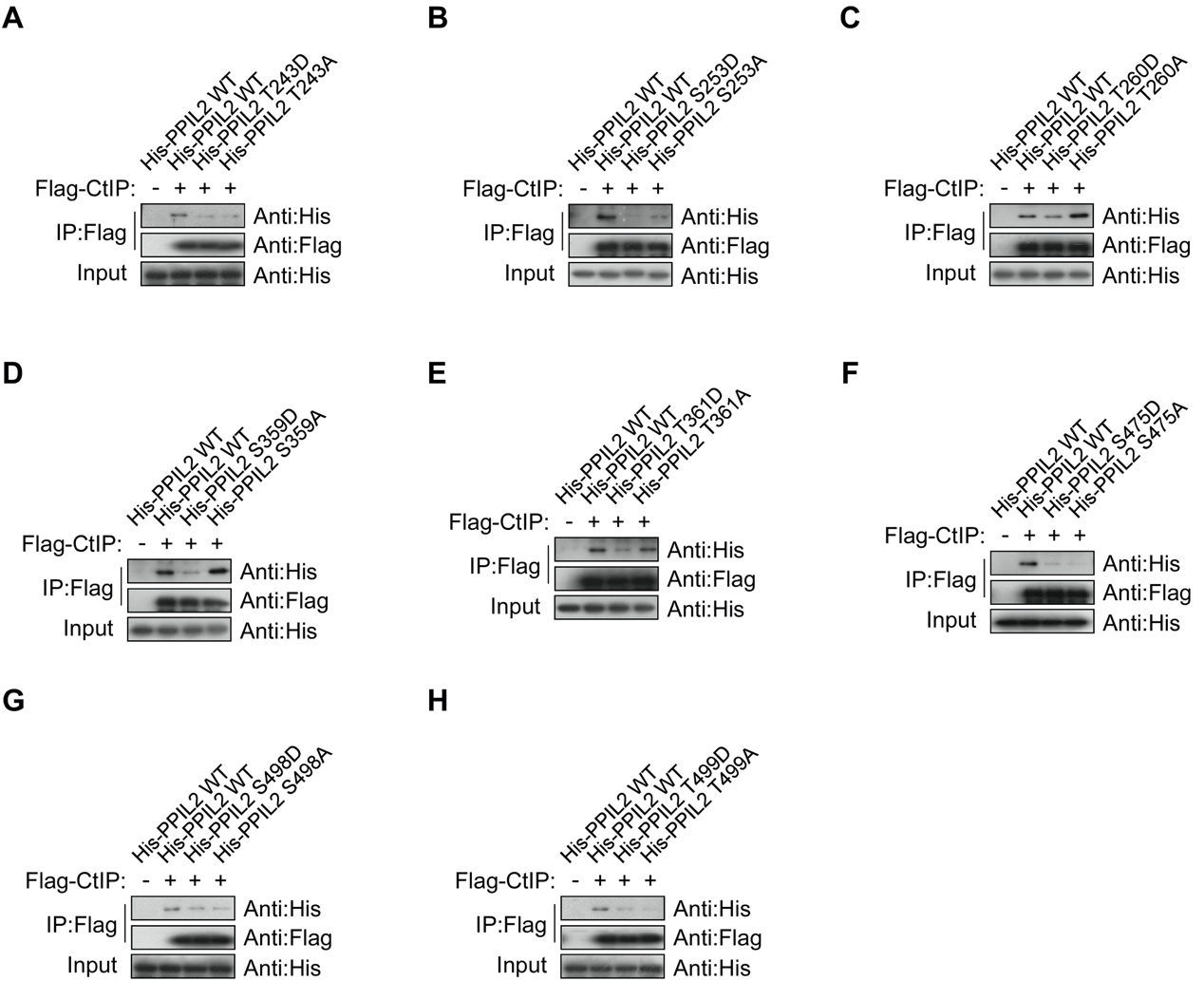


**Supplementary Figure S4.** (**A**-**H**) 293T cells were co-transfected with Flag-CtIP and His-PPIL2 single-point mutant expression constructs. 48 h post transfection, IP and western blotting with the indicated antibodies.

**Supplementary Figure S5.**


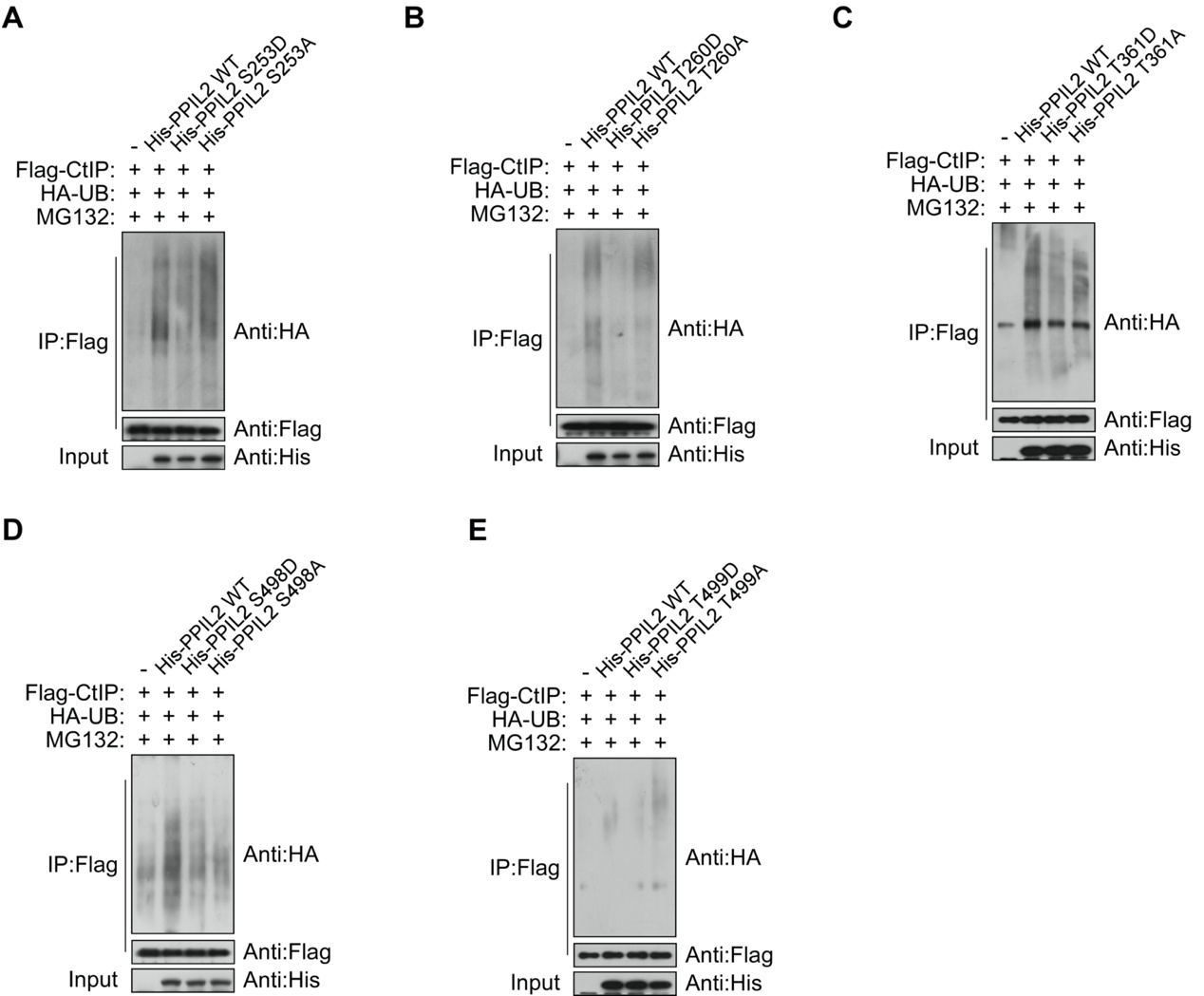


**Supplementary Figure S5.** Point-mutation of PPIL phosphorylation sites reduces CtIP ubiquitination. (**A**-**E**) 293T cells were cotransfected with Flag-CtIP, HA-Ub and His-PPIL2 single-point mutantd. 48 h post transfection, the cells were treated with MG132 (20 μM) for 3 h. The cells were then lysed in NETN buffer containing a protease inhibitor cocktail (PIC), and the cell lysate was immunoprecipitated with anti-Flag and analysed by immunoblotting with anti-HA.

**Supplementary Figure S6.**


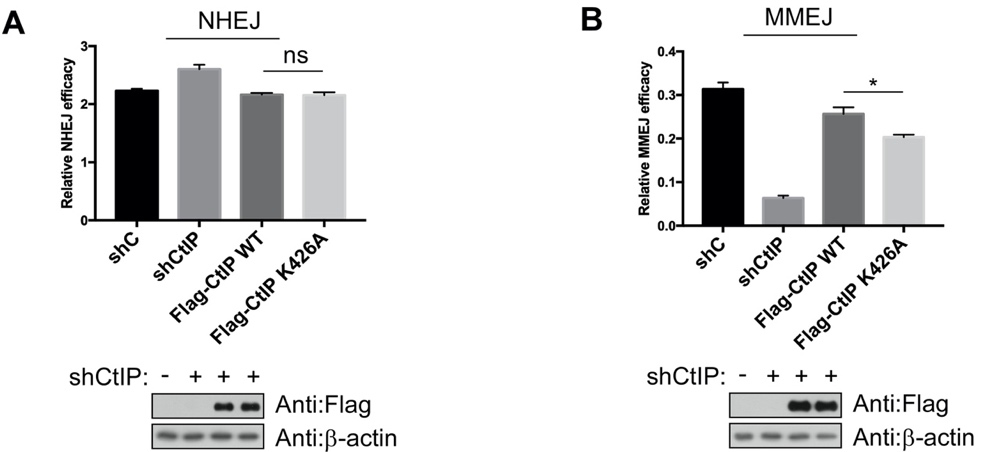


**Supplementary Figure S6.** PPIL2 inhibits the ubiquitination of PLK1. EGFP-NHEJ **(A)** and EFGP-MMEJ **(B)** assays were performed in U2OS cells stably expressing Flag-CtIP WT or K424A mutant, and U2OS cells were infected with shRNA control (shC) or shPPIL2. Western blotting shows the expression of Flag-CtIP variants. The data represent the means of three independent experiments, with error bars as SEM and P values as noted: **p*<0.05; ns: not significant.

**Supplementary Figure S7.**


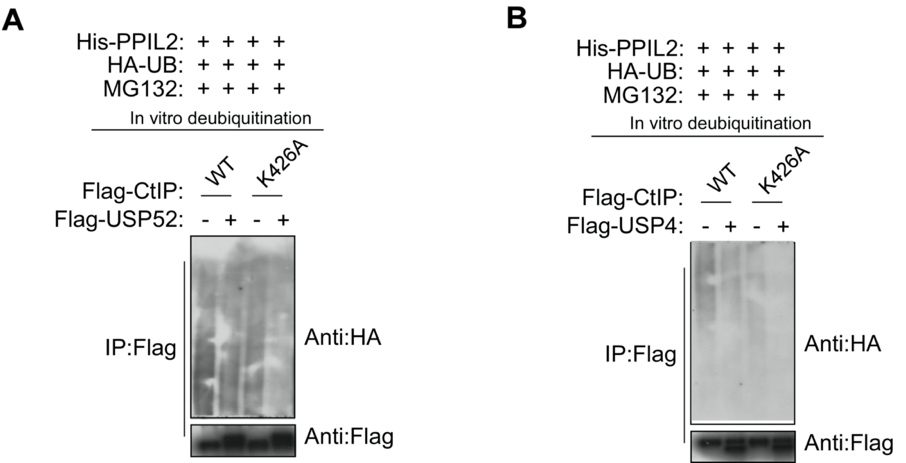


**Supplementary Figure S7.** USP52 and USP4 do not deubiquitinate CtIP *in vitro*. 293T cells were cotransfected with Flag-CtIP WT or K426A, HA-Ub and His-PPIL2 single-point mutant. 48 h post transfection, the cells were treated with MG132 (20 μM) for 3 h. The cells were then lysed in NETN buffer containing a protease inhibitor cocktail (PIC), and the cell lysate was immunoprecipitated with anti-Flag. 293T cells were transfected with **(A)** Flag-USP52 and **(B)** Flag-USP4. 48 h post transfection, IP with anti-Flag and elution with Flag-peptides (Sigma). For the in vitro deubiquitination assay, ubiquitinated Flag-CtIP WT or K426A protein with FLAG-USP52 in a deubiquitination buffer (50 mM Tris-HCl pH 8.0, 50 mM NaCl, 1 mM EDTA, 10 mM DTT and 5% glycerol) and incubated 4 h at room temperature.
